# Enhancement of Proteolytic Activity of a Thermostable Papain-Like Protease by Structure-Based Rational Design

**DOI:** 10.1371/journal.pone.0062619

**Published:** 2013-05-03

**Authors:** Sruti Dutta, Jiban Kanti Dattagupta, Sampa Biswas

**Affiliations:** Crystallography and Molecular Biology Division, Saha Institute of Nuclear Physics, Kolkata, India; Bioinformatics Institute, Singapore

## Abstract

Ervatamins (A, B and C) are papain-like cysteine proteases from the plant *Ervatamia coronaria*. Among Ervatamins, Ervatamin-C is a thermostable protease, but it shows lower catalytic efficiency. In contrast, Ervatamin-A which has a high amino acid sequence identity (∼90%) and structural homology (Cα rmsd 0.4 Å) with Ervatamin-C, has much higher catalytic efficiency (∼57 times). From the structural comparison of Ervatamin-A and –C, two residues Thr32 and Tyr67 in the catalytic cleft of Ervatamin-A have been identified whose contributions for higher activity of Ervatamin-A are established in our earlier studies. In this study, these two residues have been introduced in Ervatamin-C by site directed mutagenesis to enhance the catalytic efficiency of the thermostable protease. Two single mutants (S32T and A67Y) and one double mutant (S32T/A67Y) of Ervatamin-C have been generated and characterized. All the three mutants show ∼ 8 times higher catalytic efficiency (*k*_cat_/*K*_m_) than the wild-type. The thermostability of all the three mutant enzymes remained unchanged. The double mutant does not achieve the catalytic efficiency of the template enzyme Ervatamin-A. By modeling the structure of the double mutant and probing the role of active site residues by docking a substrate, the mechanistic insights of higher activity of the mutant protease have been addressed. The *in-silico* study demonstrates that the residues beyond the catalytic cleft also influence the substrate binding and positioning of the substrate at the catalytic centre, thus controlling the catalytic efficiency of an enzyme.

## Introduction

Proteases or proteolytic enzymes regulate various important biological processes in a living cell and they are extensively studied and well characterized [1]. They are also widely used in industry. Papain, bromelain, and ficin are the most frequently used plant cysteine proteases (clan C1A) in different industries [2]. These proteases are used in various processes such as brewing, meat softening, milk-clotting, cancer treatment, digestion and viral disorders [3]–[7]. Although the existing commercially available plant cysteine proteases have a high degree of proteolytic activity, the need for new proteases with more appealing physicochemical properties for industry are emerging [8]. In this context, we can mention that higher stability of industrial enzymes is generally considered as an economic advantage because of reduced enzyme turnover. In addition, stable enzymes permit the use of high process temperatures, which usually have beneficial effects on reactant solubility and they reduce the risk of microbial contamination [8]–[9]. However, naturally occurring thermostable enzymes generally have compromised catalytic efficiency [10]. To exploit the high catalytic efficiency at elevated temperature, efforts are made to improve catalytic efficiency in thermostable enzymes by protein engineering techniques [10]. There are two general strategies for protein engineering: 1) directed evolution by randomly mutating the gene and subsequent selection of the mutant(s) with desired properties and 2) rational design in which targeted mutations are carried out in the gene, guided by structural analyses using X-ray crystallography and molecular modelling [8], [11]–[12]. This latter approach has been proven to be straightforward for generating desired properties in enzymes, if three dimensional (3D) structures of the concerned enzymes are available.

Ervatamin-C (Erv-C), a papain-like cysteine protease from the latex of *Ervatamia coronaria* is a thermostable enzyme [13] which retains both secondary and tertiary structures and biological activity at a wide range of pH (2–12), at a high temperature (70°C) and at a high concentration of chemical denaturants. The 3D X-ray structure of Erv-C [14]–[15] determined by us reveals an extra disulfide bond, shorter loop regions and additional electrostatic interactions in the interdomain space, which are thought to be responsible for its high stability. However, kinetic studies in our laboratory revealed that enzymatic activity of Erv-C is much lower compared to other papain-like proteases [16]. The aim of this study was to enhance the catalytic activity of Erv-C retaining its stability to make it a useful biocatalyst with a potential for industrial applications.

**Figure 1:**
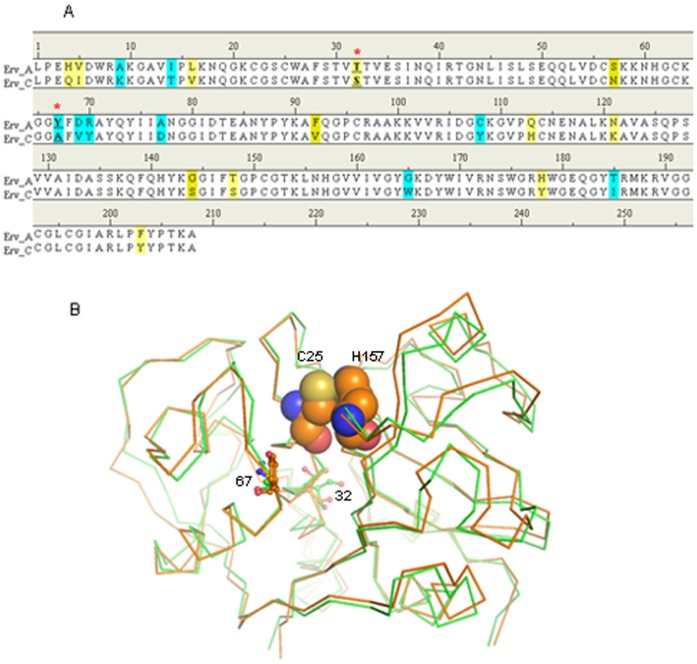
Comparison of Erv-A and -C. A. Amino acid sequence alignment of Erv-A and –C. Similar residues and mismatched residues are shaded yellow and sky blue. The mismatched residues in the catalytic clefts which are targets for mutation are indicated by red stars. **B.** Structural superposition of Erv-A and –C. The structures of Erv-A and –C are represented by Cα traces in orange and green colours respectively. The mismatched residues in the catalytic clefts are represented by ball and stick models. The catalytic dyad residues Cys25 and His 157 of Erv-A are presented as spheres.

For rational design of new mutants of Erv-C with an improved catalytic efficiency, it is important to explore the major factors affecting the catalytic properties of Erv-C and if possible to understand the correlation between the structure and catalytic efficiency of the protease. In the present study, we have used the structure of Ervatamin-A (Erv-A), another papain-like protease from the same source having ∼90% amino acid sequence identity (Fig. 1A) but with much higher catalytic efficiency compared to Erv-C [16], as a template to identify the factors responsible for higher catalytic efficiency. Two homologous 3D structures of Erv-A and Erv-C (Fig. 1B) have been compared to identify the mutations in the catalytic cleft which need to be incorporated in Erv-C to enhance its proteolytic activity. The mutants were generated and characterized experimentally which showed higher catalytic efficiency compared to wild-type. We also aimed to explore the structure-activity correlation by modelling the structure of enzyme-substrate complexes of Erv-A, Erv-C and the double mutant of Erv-C. The combined structural and experimental results demonstrate a reasonable correlation between the structure and the catalytic efficiency of the enzyme.

## Materials and Methods

### Site Directed Mutagenesis, Protein Expression and Purification

The wild-type pro-Erv-C clone in pET-28a(+) vector [17] was used for introducing the mutations. Two single mutants, S32T, A67Y (mature Erv-C numbering) and one double mutant S32T/A67Y were generated using QuickChange® Site-Directed Mutagenesis Kit (Stratagene, LaJolla, CA, USA) according to manufacturer’s protocol with the specifically designed complementary primers containing the desired point mutations (Table 1). The double mutant was constructed by extracting the DNA encoding the S32T enzyme and mutating it further with the primer for the A67Y mutation. In all cases, original unmutated template DNA was digested with Dpn-I restriction nuclease (1 h at 37°C) to remove methylated and hemimethylated parental DNA template and the mutated plasmids were transformed into *E. coli* DH10B cells. Selected colonies were grown overnight and plasmids were purified using QIAprep Spin Miniprep kit (QIAGEN, New Delhi, India). All plasmids were sequenced using the MegaBACE™ 1000 sequencing system (Amersham Biosciences, USA) to confirm the specific mutation(s) generated without additional changes.

**Table 1:** Oligonucleotides used for site-directed mutagenesis.

Mutants	Oligonucleotide primers^a^	
S32T	5′-GCTGGGCCTTCTCAACAGTT**ACC**ACCGTAGAATCTATTAACCAG-3′	Forward
	5′-CTGGTTAATAGATTCTACGGT**GGT**AACTGTTGAGAAGGCCCAGC-3′	Reverse
A67Y	5′-CATGGTTGCAAGGGAGGC**TAT**TTTGTCTATGCTTATCAG-3′	Forward
	5′-CTGATAAGCATAGACAAA**ATA**GCCTCCCTTGCAACCATG-3′	Reverse

a Underlined sequences represent the designated mutation for the target amino acid residue.

For recombinant protein production, plasmids bearing the desired mutations were transformed into *E. coli* BL21(DE3) expression strain and sequences were again verified. Mutant proteins were expressed, purified, refolded and concentrated with the same conditions as that of wild-type protein [17]. Briefly proteins expressed as inclusion bodies, which were solubilized with guanidine hydrochloride, purified by Ni-NTA affinity chromatography under denaturing conditions and refolded by dialysis method [17]. The refolded protein was concentrated by Amicon Ultra-4 (10 kDa cut-off) for further studies.

### Proteolytic Activity Assay and Kinetic Analysis

#### a) Zymogen activation

The purified refolded mutant pro-enzymes (10–20 µg) were activated by using cysteine (20 mM) as an activator in 50 mM Na-acetate buffer pH 5.00 at 60°C like wild-type protein, except that the time of activation had to be re-optimized.

#### b) Optimal pH analysis

Each enzyme has a pH range in which it shows maximum rate of reaction. This maximum is known as the pH optimum of the enzyme. The purified refolded mutant pro-enzymes (10–20 µg) were activated and pH optima of mutant proteins were determined against the substrate azocasein at different pH values ranging from 4.5 to 8.0 as described before [18]. The wild- type was also used for comparison.

#### c) Gelatin gel zymography and Azocaesin assay

Substrate gel zymography using 0.1% gelatin as a substrate was used to demonstrate the protease activity of recombinant refolded mutant proteases (S32T, A67Y and S32T/A67Y) and wild-type protease using a protocol described previously [17]. After the activation of mutant pro-enzymes as described above, the specific activity of the recombinant mutants along with the wild-type protease was determined using substrate azocasein [18]. One enzyme unit was defined as the amount of protease required to release 1 µg of soluble azopeptides/min. The specific activity was the number of units of activity per milligram of protein.

#### d) Kinetic analyses with peptidyl pNA as substrate and inhibition with E-64

Kinetic parameters *K*_m_ and *k*_cat_ was measured at 298 K by continuously monitoring the release of p-nitroaniline (pNA) from N-benzoyl-Phe-Val-Arg-pNA (Sigma, Missouri, USA) at 410 nm using an extinction coefficient of 8800 M^−1^ cm^−1^ for pNA [19] on a UV/Vis spectrophotometer (Nicolet Evolution 100; Thermo Electron Corporation, Rockville, MD, USA). Complete conversion of pro-Erv-C to active mature enzyme was optimized for each mutant as described above and the activated form of the mutant were used for this assay. The optimum concentrations of the mutant enzymes were also standardized. The *K*_m_ and *V*_max_ values for each enzyme-substrate combination were estimated by nonlinear fitting of the Michaelis–Menten saturation curve using the software Graphpad PRISM (http://www.graphpad.com/prism). The *k*_cat_ value was determined by using the equation *k*_cat_ = *V*_max_ / [E]_T_ where [E]_T_ is the total concentration of the active enzyme, the values of which were measured by active-site titration with the irreversible inhibitor, E-64 (the irreversible inhibitor) using the above mentioned pNA containing substrate.

IC_50_ values were measured for E-64. Concentration of the substrate was kept constant at levels below the apparent *K*_m_. E-64 was added in increasing concentrations to the activated pro-enzyme solutions and incubated for 2–5 min until the residual activity reached zero. Residual activity (Δ*A*_410 nm_min^−1^) was calculated with respect to the full activity of the enzyme that was carried out without any inhibitor [18] against the substrate. The residual activity versus inhibitor concentration was plotted and the 50% inhibitory concentrations (IC_50_) of E-64 for these mutant enzymes were determined from this plot.

### Optimal Temperature of Activity and Thermostability Analyses

Each enzyme has a temperature range in which a maximal rate of reaction is achieved. This maximum is known as the temperature optimum (*T*_opt_) of the enzyme. The purified refolded mutant pro-enzymes (10–20 µg) were activated and *T*_opt_ of mutant proteins were determined against the substrate azocasein as described before [18]. The wild-type was also used for comparison.

The kinetic thermal stability of the mutant proteins along with the wild-type protein was determined. The purified refolded mutant pro-enzymes (10–20 µg) were incubated over a temperature range of 40°–80°C at 5° intervals for 10 min each in a temperature controlled water bath and then immediately chilled in an ice bath. Mutant pro-enzymes were activated as described in the previous section. The enzyme activity was measured using the substrate azocasein and *T*_max_ was determined as described previously [18] for wild-type protein. The temperature of incubation at which the enzyme showed maximum activity was considered as *T*_max._

### Molecular Modeling Studies

#### a) In-silico generation of mutant enzymes

For generation of S32T/A67Y mutant of Erv-C, crystal structure of wild-type Erv-C (pdb_id: 2pns) was used as a starting model. Initially all the ligand atoms other than water molecules were deleted from the crystal structure. All molecular modeling simulations were performed by Discovery Studio 2.5 (Accelrys, USA) with CHARMm forcefield and CFF partial charges were used in simulations. The target residues were mutated (S32T and A67Y) and the lowest energy rotamer conformations were chosen. The atomic positions were minimized by 200 cycles steepest descent (SD) with the following two conditions, 1) the back-bone of the protein was kept fixed and 2) presence of a 35 Å water sphere generated around the center of mass of the protein. All water molecules were subsequently removed from the resulting structure and the protein molecule was solvated using ‘Explicit Periodic Boundary’ option where the system was neutralized and solvated in a rectangular box of TIP3P water molecule with a minimum solute-wall distance of 7.0 Å. The entire system was then optimized initially by 100 cycles SD and then by conjugate gradient (CG) till the derivative reached 0.01 kcal/mol/Å using the Particle Mesh Ewald approach for the evaluation of the electrostatic energy term with 12 Å distance cut off. A similar protocol was also applied for wild-type Erv-C (pdb_id: 2pns) and Erv-A (pdb_id: 3bcn) structures to relax the crystal packing force for comparison with the mutant in a similar platform.

#### b) Enzyme-substrate modeling

The substrate N-benzoyl(P4)-Phe(P3)-Val(P2)-Arg(P1)-↓-pNA(P1′) chosen for this study is the same which was used in experimental kinetic analyses of the enzymes. The substrate was generated and optimized in vaccuo for 100 steps SD by Discovery Studio.

The structures of Erv-C and Erv-A in complex with covalently attached inhibitor E-64 (pdb_ids: 2pre and 3bcn) were used as a template for docking the substrate at the catalytic cleft of Erv-C, Erv-A and double mutant of Erv-C (S32T/A67Y). E-64 is covalently bound to active site cysteine in a reverse orientation than that of a substrate and the leucyl moiety of E-64 occupies the S2 pocket of the enzyme. The substrate was manually fitted at the active site cleft of enzymes such that the carbonyl oxygen atom of the scissile peptide bond points towards the oxyanion hole for a productive nucleophilic attack. The side chains of P1, P2 and P3 were placed like those in the E-64 in complex structures. P4 and P1′ moieties were manually adjusted in the catalytic cleft considering the charge complementarities, hydrophobicity and by avoiding short contacts. Finally delicate manual adjustments were performed to gain maximum number of hydrogen bonds between the back-bone of the substrate and the enzymes. The resulting complexes thus generated were used for minimization and simulation. A distance constraint of 3.0 Å between catalytic Cysteine SG atom and carbonyl carbon atom of the scissile peptide bond of the substrate molecule was used in all subsequent calculations. A spherical water shell of 30 Å radius was generated with the centre of mass of the substrate for each complex and a minimization of 100 cycles SD was performed keeping the complex molecule fixed to allow the water molecules to adjust. Now the entire complex was minimized initially by 500 cycles SD and 500 cycles CG by using harmonic restrain on the back-bone. A short molecular dynamics by ‘standard dynamics cascade’ protocol was applied with default parameters except that the same constrain/restrains for each complex as discussed above were applied. The system was heated to 300K in 20 ps followed by an equilibration of 100 ps at 300K. Finally a production run was done for 1 ns (Fig. S1) where conformations were trapped in 1 ps interval. Initial 100 conformations of the production run were further minimized by 100 cycles SD without any constrain/restrain. These 100 low energy poses of the docked substrate in each case were used for analysis.

## Results and Discussion

### Expression and Purification of the Mutant Enzymes

**Figure 2:**
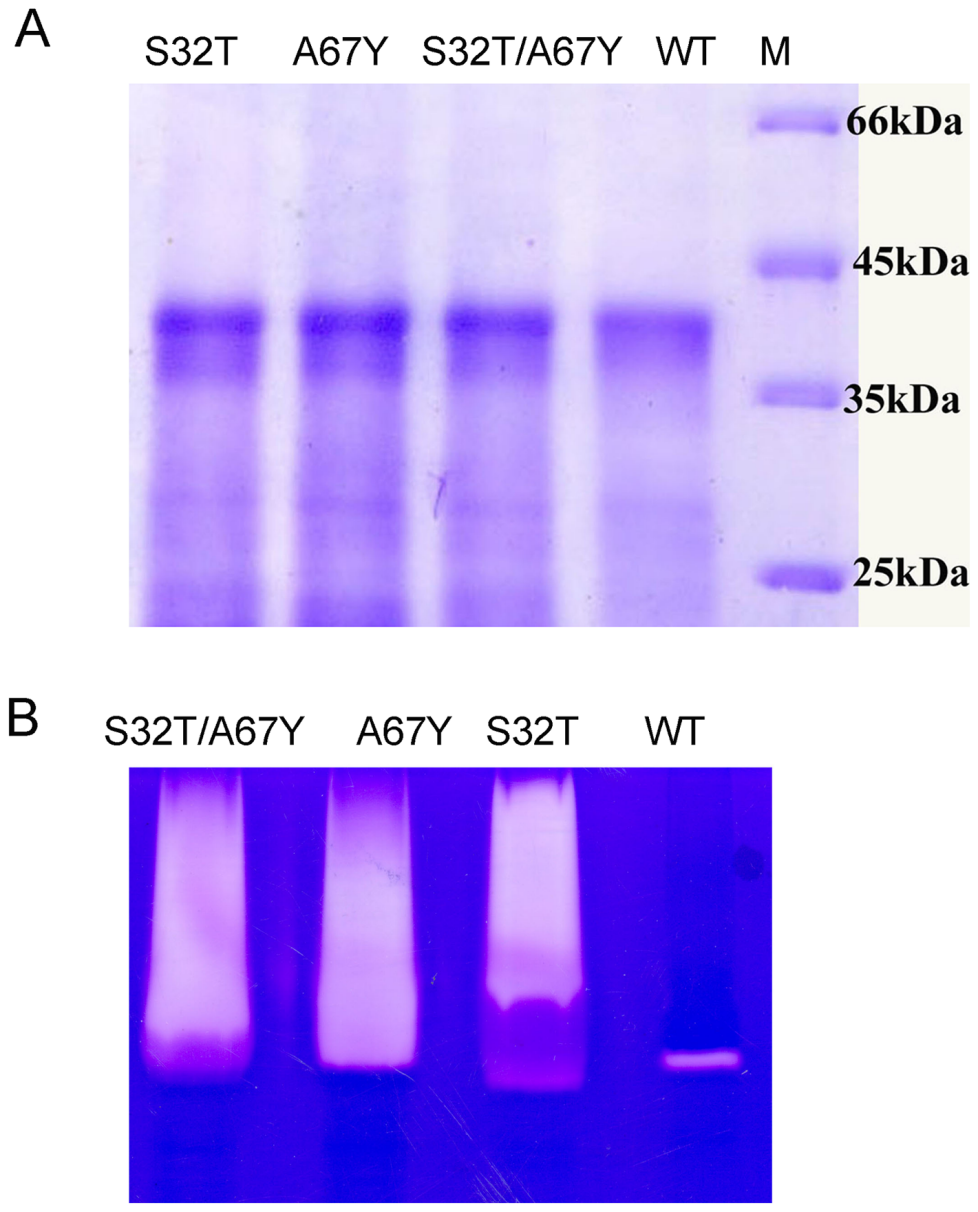
SDS–PAGE analysis and gelatin gel zymography. A. Purified and refolded pro-enzymes of mutants and wild-type were analyzed in 15% SDS-PAGE; M denotes Molecular mass markers. **B.** Gelatin gel assay of the activated mutants (∼15 µg) and wild-type enzymes.

Purification and refolding of the recombinantly expressed mutant enzymes were confirmed by SDS-PAGE (Fig. 2A). The conditions, efficiency of refolding and the yield were similar for all the mutant enzymes and the wild-type [17]. The proteolytic activities of the refolded proteins were verified by zymography (Fig. 2B).

### Enzymatic Activity of the Mutant Enzymes

#### a) Zymogen activation

**Figure 3:**
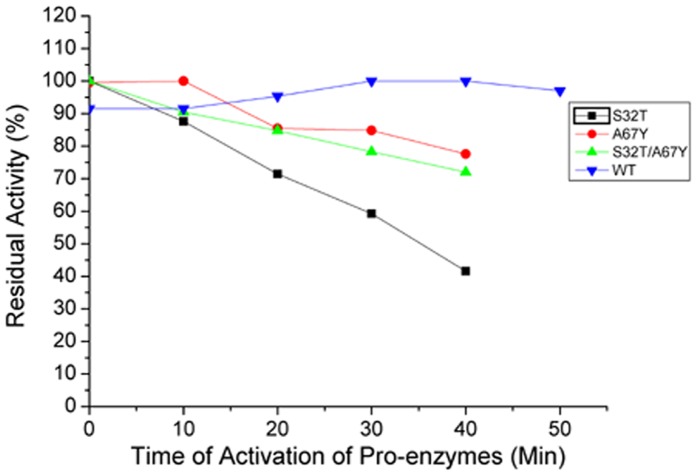
Time course of activation of pro-enzymes to the mature and active form of the wild-type and the mutants of Erv-C. Aliquots of purified pro-enzymes (10–20 µg) were treated for activation for 0 to 50 minutes to convert into their respective mature forms and the percentage of residual enzyme activities were determined with respect to the maximum activity using an azocasein assay, as described in Materials and methods.

The mutant pro-enzymes could be activated by using the same activator, buffer (20 mM cysteine in 50 mM Na-acetate buffer pH 5.00) and temperature (60°C) like wild-type Erv-C [17]. However, the time of activation of all the three mutant pro-enzymes significantly differs from the wild-type. S32T and S32T/A67Y could be activated by giving a short heat shock for 30 sec at 60°C while for A67Y, an incubation of 10 min at 60°C is required to get maximum activity. The activity of S32T sharply decreases with time while for other two mutants, activities decrease slowly. In contrast, wild-type required 30–40 min for reaching maximum activity and more than 90% activity is observed (Fig. 3) during the time range of 20–50 min.

#### b) Optimum pH of activity

**Figure 4:**
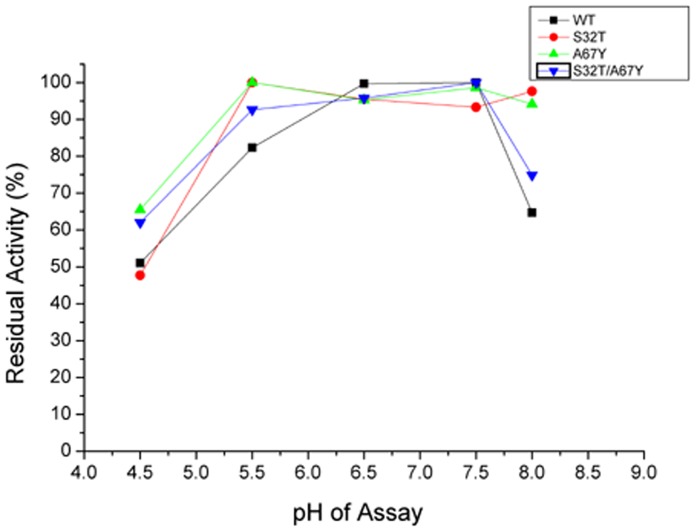
Determination of optimum pH of activity of the wild-type and the mutants of Erv-C. Purified pro-enzymes (10–20 µg) were converted to their respective mature forms and the percentage residual enzyme activities were determined with respect to the maximum activity using an azocasein assay at different pH, as described in Materials and methods. Each data point is an average of three independent experiments having similar values.

Mutant enzymes show high proteolytic activity at neutral pH range (6.5–7.5) like wild-type (Fig. 4). All the three mutants have a broad range of pH optima (90–100% activity in the pH range 4.5–7.5) which can be of advantage for large scale production.

#### c) Gelatinolytic and caseinolytic activity

Refolding efficiency was checked by gelatin gel assay (Fig. 2B) where mutant enzymes show high protease activity compared to wild-type enzyme.

All the mutants S32T, A67Y and S32T/A67Y show higher caseinolytic activity than wild-type (Table 2). Notably specific activity for A67Y mutant against azo-casein is almost 2.4 times higher than wild-type.

**Table 2:** Kinetic constants using the substrate N-benzoyl-Phe-Val-Arg-pNA. Specific activity using azocasein and IC_50_ value for the inhibitor E-64.

	*k*_cat_(s^−1^)^a^	*K*_m_ (µM)^a^	*k*_cat_/*K*_m_ (s^−1 ^mM^−1^)	Specific activity^b^ (U/mg)	IC_50_ against E-64 (nM)
S32T	2.23±0.15	149.90±17.26	14.87±1.97	73.63±1.52	213.75
A67Y	2.65±0.13	176.90±14.11	14.75±1.38	86.95±1.32	250.75
S32T/A67Y	1.43±0.10	96.87±12.43	14.76±2.12	63.11±1.70	171.17
Wild type	0.23±0.06	127.30±48.46	1.80±0.81	36.78±1.70	349.00

a Standard errors were calculated based on nonlinear fitting of the Michaelis–Menten saturation curve using the software Graphpad PRISM (http://www.graphpad.com/prism).

b Each value of specific activity of each enzyme is a mean of three independent experiments ± SD.

#### d) Kinetic analyses and inhibition with E-64

To measure the kinetic parameters of the mutant enzymes, synthetic substrate N-benzoyl-Phe-Val-Arg-↓-*p-*nitroanilide (pNA) has been used at various concentrations and the liberated pNA were measured chromogenically. The *k*_cat_ and *K*_m_ values are summarized in Table 2. The *k_cat_* values were influenced in all the three mutants of Erv-C which increased almost 10-fold in each case. However, the effect of the mutation on *K*_m_ values was small and increased slightly for the two single mutants whereas it was somewhat decreased for the double mutant. From this result, it appears that the particular mutations at positions 67 and 32 did not influence the affinity of the enzyme towards this substrate, but did affect the catalytic activity. The changes in the conformation of active sites of the mutant enzymes due to mutation(s) may be the possible reasons for alteration of the catalytic activity which has been explained from the molecular modeling studies in a following section.

To determine the effect of cysteine protease specific inhibitor E-64 on the activities of the mutants, the IC_50_ values were measured (Table 2) and compared with wild-type. The sensitivity of the double mutant (S32T/A67Y) for E-64 is 2 times higher than that observed for wild-type. The other two single mutants are also more sensitive compared to wild-type.

### Effect of Mutations on Optimal Reaction Temperature and Thermal Stability

**Figure 5:**
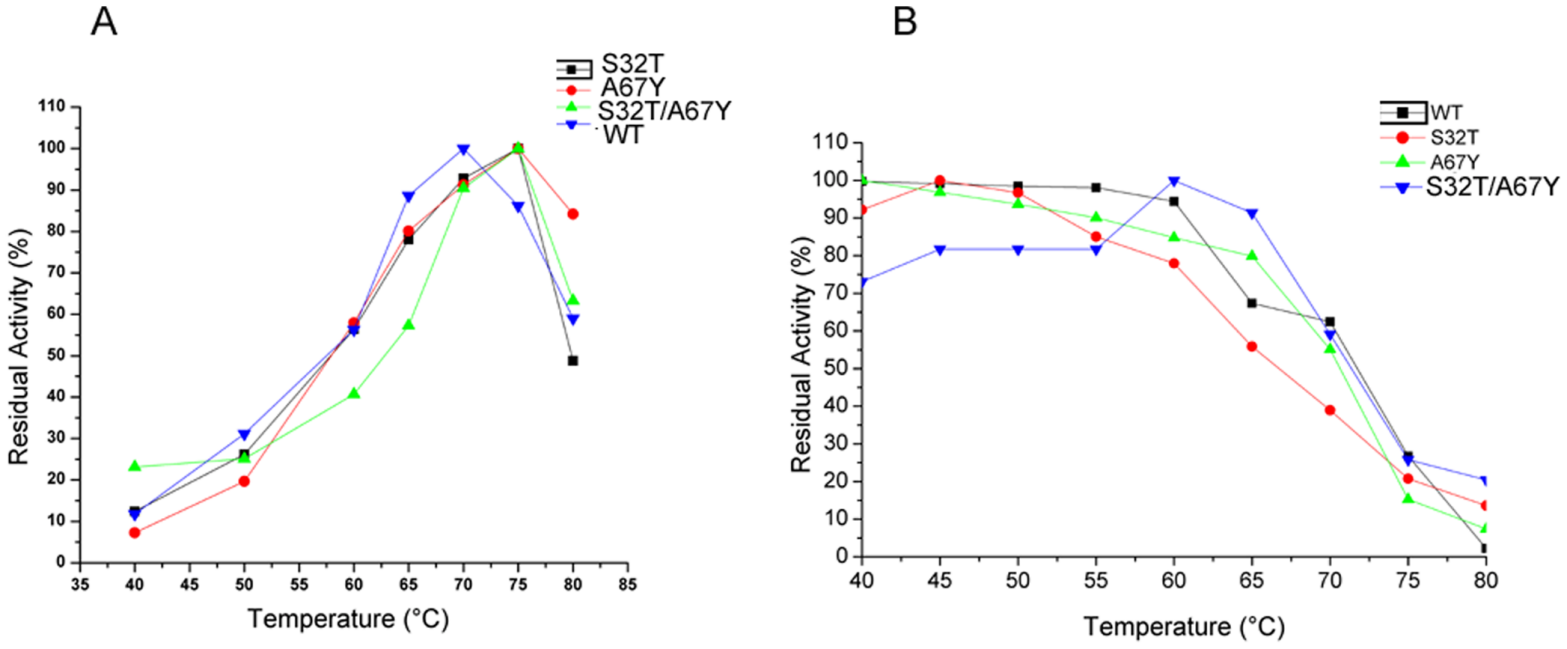
Analyses of thermal stability of the wild-type and the mutants of Erv-C. A. Determination of optimum temperature of activity (*T*_opt_) of the wild-type and the mutants of Erv-C. Purified pro-enzymes (10–20 µg) were converted to their respective mature forms and the percentage residual enzyme activities were determined with respect to the maximum activity using an azocasein assay at different temperatures as described in Materials and methods. **B.** Effect of temperature on activity of the wild type and the mutants of Erv-C. Each purified pro-enzyme (10–20 µg) was treated for 10 min at different temperatures followed by activation of the pro-enzymes to their respective mature forms. The percentage residual enzyme activities (at each temperature) were determined with respect to the maximum activity using an azocasein assay. Each data point is an average of three independent experiments having similar values for both the graphs.

Operational stability of a biocatalyst is an important aspect for its use in different applications, particularly those which need large scale production. Enhancement of proteolytic activity of S32T, A67Y and S32T/A67Y mutants makes them promising enzymes for potential application in industry. To investigate whether the mutant enzymes with enhanced catalytic activity retain their thermal stability like wild-type, we measured and compared optimum temperature of activity (*T*_opt_) (Fig. 5A) and temperature of maximum activity (*T*_max_) (Fig. 5B) (Table 3). As shown in Figure 5A, the *T*_opt_ analyses suggest that proteolytic activities of all the mutants are in the range of 65°–70°C which are similar to that of the wild-type. Temperatures of maximum proteolytic activity (*T*_max_) for S32T, A67Y and S32T/A67Y were 45°C, 40°C and 60°C respectively (Table 3). These findings indicate that the mutant enzymes in general retain thermal stability almost like their wild-type counterpart.

**Table 3:** Kinetic stabilities.

	*T*_max_(°C)	*T*_50_(°C)^a^	*T*_opt_(°C)
S32T	45	67	70
A67Y	40	71	70
S32T/A67Y	60	72	70
Wild type	45	72	70

a *T*_50_ was expressed as the temperature of incubation at which the enzyme showed 50% of maximum activity.

### Comparison of Energy Minimized Structures of Erv-C and –A and Double Mutant of Erv-C (S32T/A67Y)

Catalytic cleft of papain-like cysteine proteases is at the interface of the two domains. Residues from two domains form the catalytic dyad (Cys^–^His^+^) (Fig. 1B) and substrate binding subsites S1′, S1, S2 and S3 [20]–[21]. It is well established that S2 forms a deep pocket which determines the specificity of this family of proteases [20]. Therefore the residues lining the S2 subsite cleft are important in forming the size, shape and charge distribution of the subsite which essentially determines the specificity for the residue at the P2 position of the substrate. The stability of the zwitterionic form of the catalytic dyad (Cys^–^His^+^) is responsible for the nucleophillic attack to the carbonyl carbon atom of the scissile peptide bond [21]. The most important contribution in stabilizing the zwitterions comes from the long central α-helix to which the catalytic Cys belongs [21]. The polarizing effect originating from the concerned helix facilitates the transfer of the proton from the catalytic Cys present at the N-terminus of the helix to the His of the dyad [21]. The length and sequence of the constituent residues of the poly-peptide substrate or stretch of polypeptide of a protein substrate accessible to the protease catalytic cleft are also important for anchoring and subsequent positioning of the peptide bond of the substrate for cleavage. The overall catalytic efficiency of a protease is therefore dependent on the combined factors described above.

**Figure 6:**
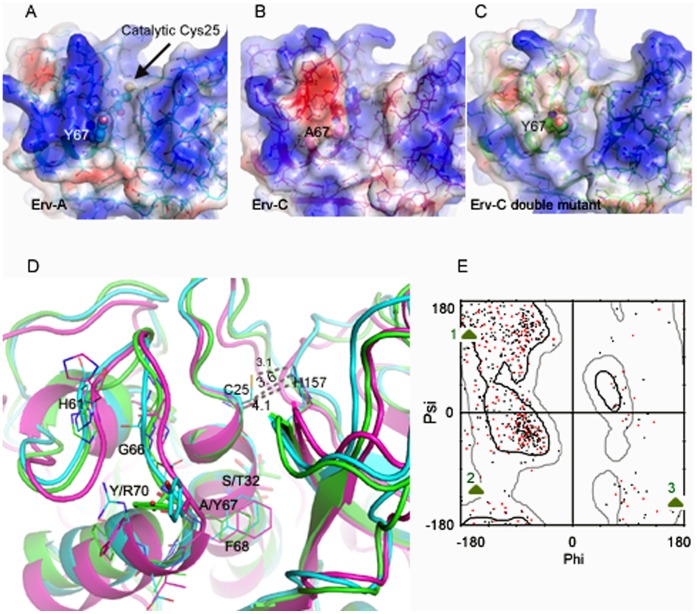
Comparison of three dimensional structures of Erv-A, Erv-C and double mutant of Erv-C (S32T/A67Y). **A**, **B**, and **C**. Surface presentation of Erv-A, Erv-C and double mutant of Erv-C. The residues in positions 32, 67 and catalytic cysteins are presented in ball and stick model. **D**. Overlay of the three dimensional structures of the three enzymes; sky-blue, magenta and green colored cartoons are for Erv-A, Erv-C and double mutant of Erv-C. The important residues are labeled and represented in stick model. Distances of the catalytic dyad (C25SG and H157ND1) of the three enzymes are marked. **E**. Ramachandran plot highlighting G66 residues in three enzymes (1:Erv-A, 2: Erv-C and 3:Erv-C double mutant). The red colored points are for the double mutant of Erv-C.

It is established that both native Erv-A and Erv-C, purified from the latex of the plant, prefer a branched hydrophobic side chain (Val or Leu) at the P2 position of a substrate [16]. Of the five residues (Tyr67, Phe68, Ala131, Leu155 and Leu201) forming the S2 subsite cleft of Erv-A one substitution (Tyr67 is replaced by Ala67) is observed in Erv-C [16]. The side chain of Tyr67 in Erv-A points towards the interface of S2 and S3 subsites, providing tight packing and a favourable hydrophobic environment for P2 position of a substrate at the S2 subsite cleft [16] (Fig. 6A). Due to Tyr67→Ala67 replacement, the S2 cavity of Erv-C has a wider opening than that of Erv-A and as a result lacks the proper environment to bind and fix a hydrophobic side chain (P2) of a substrate [16] (Fig. 6B).

In Erv-A, Thr32 belongs to the same helix where Cys25 is situated and T32OG orients towards the α-helical axis (Fig. 1B) forming H-bonds with the back-bone atoms. In Erv-C, Thr32 is replaced by a Ser32 and S32OG points away from the helix axis. The H-bond of T32OG with back-bone atoms in Erv-A are thought to be responsible for enhanced dipole-moment resulting in a stable zwitterionic form of the catalytic dyad which is a pre-requisite for higher catalytic efficiency.

The objective of this study is to enhance stability of the zwitterionic form of the catalytic dyad (Cys^–^His^+^) and to create an environment in S2 pocket of Erv-C which will be similar to those of Erv-A by replacing Ser32→Thr32 and Ala67→Tyr67 respectively. In the double mutant (S32T/A67Y) of Erv-C which has been generated in this study, the catalytic efficiency (*k*_cat_/*K*_m_) is ∼8 times higher than wild-type Erv-C. We generated a model structure of the double mutant of Erv-C *in silico* for this study (Fig. 6C) to gain a structural insight of the observed difference in catalytic efficiency at the molecular level. The structural superposition of Erv-A, Erv-C and Erv-C (S32T/A67Y) double mutant was performed for comparison (Fig. 6D). The side-chain rotamer conformations of both the residues S32T and A67Y in the double mutant are different from those in Erv-A structure. The aromatic benzene ring of Tyr67 (A67Y) in the mutant is almost perpendicular to that in Erv-A. Therefore A67Y in Erv-C double mutant can not form a well defined boundary wall at the S2 pocket like it forms in Erv-A (Fig. 6C and 6A). This difference in orientation of Tyr67 (A67Y) side-chain in the double mutant of Erv-C may be due to the presence of a neighboring Tyr70 residue which in fact is an Arginine in Erv-A (Fig. 6C and 6A). The Tyr67/Tyr70 combination in Erv-C double mutant has two important effects on the structure; i) Phe68 side-chain shifts away from the S2 pocket (Fig. 6D), diminishing its contribution for S2-P2 interaction to some extent and ii) carbonyl oxygen at Gly66 cannot adopt similar orientation like that in Erv-A (Fig. 6D). These two differences in Erv-C double mutant compared to Erv-A may have long range (∼10 Å) influence on the orientation of T32OG responsible for enhancing dipole moment as mentioned above. It is postulated that G66O of Erv-A helps in positioning T32OG in proper orientation to make H-bonds with back-bone atoms of the helix to which it belongs (Fig. 6D). In comparison, G66O of Erv-C double mutant points away from the catalytic cleft (Fig. 6E) reducing the effect on T32OG. Simultaneously, shifting of Phe68 side-chain in Erv-C double mutant reduces further its long range hydrophobic effect on T32CG and T32CB atoms and as seen in Fig. 6D, T32OG moves slightly away from the helix, unable to make any strong H-bond with the back-bone atoms like it does in Erv-A, reducing thereby its effect on the dipole moment of the helix. So we can summarize that side chains of both the residues S32T and A67Y in Erv-C double mutant do not have similar orientation like Erv-A and thus their effect on the activity can be thought to be compromised. The distance between the catalytic dyad forming atoms Cys25SG and His157-ImNH in Erv-C, Erv-C double mutant and Erv-A are 4.1, 3.6 and 3.1 Å respectively (Fig. 6D) also indicating that in the mutant structure the stability of the catalytic dyad has increased but not as much as that of Erv-A. Another significant observation found in Erv-A and Erv-C, which may influence substrate binding, is the difference of surface charges in the L-domain at the vicinity of the catalytic cleft (Fig. 6A and 6B). Erv-C contains negative charge in this region because almost all back-bone carbonyl oxygen atoms of 59–66 stretch points towards the surface. On the other hand, Erv-A contains a strong positive charge due to presence of the side chain of Arg70, pointing the side-chain of His61 and back-bone N atoms of 59–66 stretch (3 of which are Gly) to this region. However the strong negative charge is diminished in Erv-C double mutant compared to its wild-type counterpart due to presence of the hydrophobic Tyr67(A67Y) residue, pointing the side-chain of His61 to this region and reorientation of the back-bone atoms of 59–66 stretch (Fig. 6C).

### Enzyme-substrate Models

**Figure 7:**
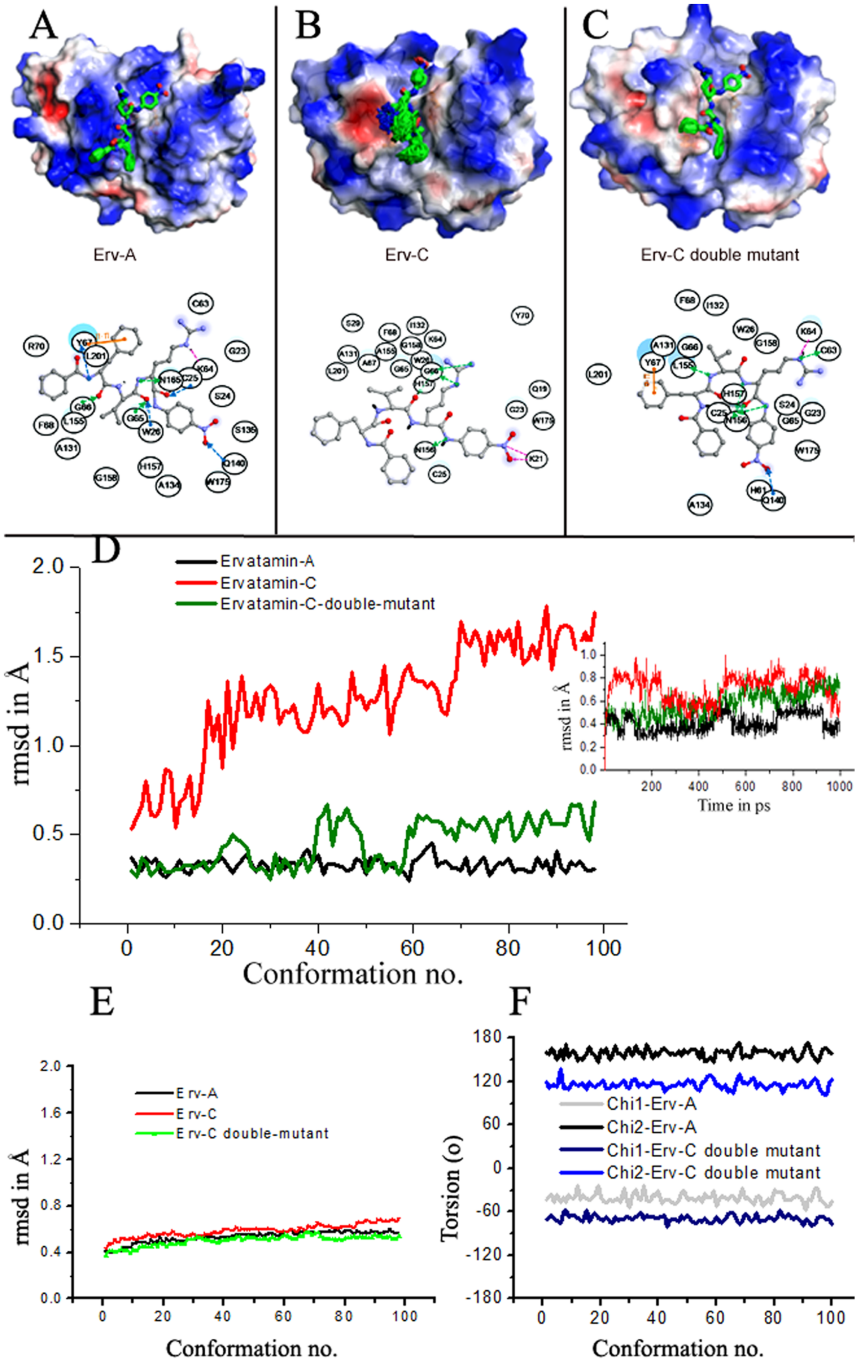
Substrate docked at the active site cleft. Overlay of 100 poses (generated after minimization of initial 100 conformations of MD trajectory) of the substrate (N-benzoyl-Phe-Val-Arg-↓-pNA) docked at the active sites of **A**. Erv-A, **B**. Erv-C and **C**. double mutant of Erv-C. Lower panels of Figures **A**, **B** and **C** are corresponding schematic representations of substrate interactions as observed in the lowest energy model of each of the enzyme-substrate complexes. **D**. Root mean square deviations (rmsd) in Å of the substrate for 100 poses as mentioned above. The rmsd of the same in the entire 1 ns trajectory has been shown in the inset figure. E. The rmsd in Å of the main chain of the three enzymes for the minimized initial 100 conformations. **F**. The side-chain torsion angles of Tyr67 in Erv-A and in the double mutant of Erv-C for 100 conformations.

Substrate residues flanking the scissile bond are major determinants for site-specific cleavage by proteases [22]. They are generally defined as Pn–P1 (unprimed) for the residues N-terminal to the scissile peptide bond and P1′–Pn′ (primed) for the residues C-terminal to the scissile bond [23]. To understand the binding and hydrolysis of peptides by Erv-A, Erv-C and the double mutant (S32T/A67Y) of Erv-C, we simulated the binding of the substrate N-benzoyl(P4)-Phe(P3)-Val(P2)-Arg(P1)-↓-pNA(P1′) in the active sites of these proteases (Fig. 7A, 7B, and 7C). The reason for choosing this substrate was that it had been used for our experimental enzyme kinetic analysis so that the observations from the modeling studies can be correlated with the enzyme kinetic data. The substrate binds to Erv-A tightly and all the 100 binding conformers are stable at the catalytic cleft with low root mean square deviation (rmsd) values (Fig. 7A and 7D). In comparison, the substrate, docked in the catalytic cleft of Erv-C, shows conformational flexibility with high rmsd values (Fig. 7B and 7D). For the double mutant, the flexibility of the substrate is restricted compared to Erv-C, however rigidity is not achieved as that of Erv-A (Fig. 7C and 7D). The main-chain rmsd values (Fig. 7E) are low (0.4–0.6 Å) in all the three enzymes indicating structural stability for all of them. The side-chain of Tyr67 of Erv-A orients towards S2–S3 cleft, forming hydrophobic and л-л interactions with Val(P2) and Phe(P3) of the substrate respectively (Fig. 7A). Even though Erv-C double mutant has the same residue at 67, its orientation is different, providing less contribution towards binding of Val(P2) and Phe(P3) of the substrate unlike Erv-A. The orientation of Tyr67 in both the enzymes is consistent in all 100 conformations (Fig. 7F). Thus the presence of Tyr67 is not only important, its orientation also plays a crucial role suggesting that the residue behaves as a hydrophobic lock of the P2/P3 residues of the substrate in the S2/S3 pockets with maximum effect for a particular orientation as is found in Erv-A. It is known that the surface charge is also important for substrate recognition. As is mentioned in the previous section, Erv-C contains a strong electronegative surface near S1 site which pulls the guanidium group of Arg(P1) side-chain of the substrate (Fig. 7B). Because of the presence of negative surface charge near S1 subsite along with absence of Tyr67 at S2/S3 subsite in Erv-C, the substrate can not fix itself at the catalytic cleft and the position of the carbonyl O atom of the scissile bond is flexible compared to that of Erv-A reducing the propensity for a nucleophillic attack by the catalytic Cysteine. However, due to mutations in Erv-C double mutant the intensity of negative charge near S1 site is reduced and Arg(P1) side-chain points outward to the solvent. Moreover presence of Tyr67 in the double mutant, whatever is the side chain orientation, has some hydrophobic effect on binding of Val(P2) and Phe(P3) which reduces the flexibility of the substrate at the catalytic cleft. This effect finally helps in positioning the scissile bond of the substrate in proper position between the catalytic dyad and the carbonyl oxygen atom of the scissile bond in the oxyanionic hole of the enzyme, enhancing propensity of nucleophillic attack by the catalytic Cysteine more than wild-type Erv-C. The calculated interaction energies between enzymes and substrate of the complexes (Erv-C: −168.07 kcal/mol, Erv-C double mutant: −79.97 kcal/mol and Erv-A: −46.63 kcal/mol) do not correspond to the experimental catalytic efficiencies (*k*_cat_/*K*_m_). The electrostatic interaction between P1(Arg) and Erv-C surface charge contribute to the interaction energy, though this interaction do not allow in taking proper position of the P1′-P1 scissile peptide bond for nucleophillic attack by the enzyme. This may be a possible reason for lower catalytic efficiency of Erv-C inspite of higher interaction energy between the enzyme and the substrate.

## Conclusion

In this study, catalytic activity is enhanced in two single mutants (S32T and A67Y) and in a double mutant (S32T/A67Y) of a thermostable papain-like cysteine protease Erv-C without compromising its thermal stability by using a structure-based site-directed mutagenesis approach. Subsequent molecular modeling of the structures of Erv-C, Erv-C double mutant (S32T/A67Y) and Erv-A (the template) and their complexes with a substrate demonstrate detailed structure–function relationships in the light of enhanced activity of the mutant of Erv-C. A plausible explanation for higher and lower activity of Erv-A and Erv-C and the fact that Erv-C double mutant (S32T/A67Y) did not achieve the activity of its template native Erv-A, could also be presented from this study. The findings of this work corroborate the crucial effects of the presence of Tyr67 and Thr32 and their side-chain orientation on the enzymatic behavior of this family as well as the importance of the region surrounding the catalytic cleft in Erv-C. The study clearly demonstrates that the kinetic performances of the enzymes are not restricted only to the engineering (SDM) of the amino acids of the catalytic cleft but is also intimately related to their environment (surroundings). This study also shows that theoretically calculated binding energy of the enzyme and a substrate does not always correspond to the experimental catalytic efficiency of the enzyme towards the substrate. We have thereby gained considerable insight into the mechanistic features that control the proteolytic activity of a papain-like cysteine protease.

## Supporting Information

Figure S1 **Variation of A. potential energy and B. temperature in molecular dynamics trajectories.**
(JPG)
