# Supplementary figures and images for: Enhancement of Proteolytic Activity of a Thermostable Papain-Like Protease by Structure-Based Rational Design
Source: PLoS One. 2013 May 3;8(5):e62619. doi: 10.1371/journal.pone.0062619 (PMC3643963; doi:10.1371/journal.pone.0062619)

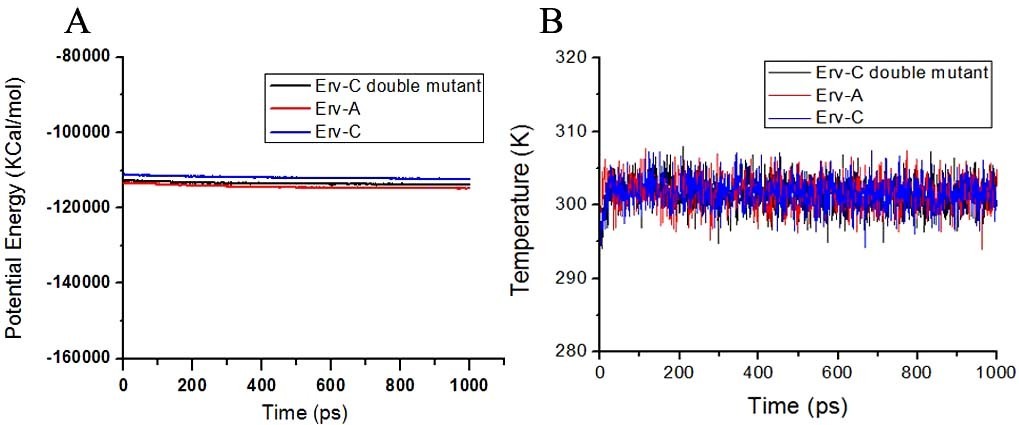

Supplement: Figure S1 — Variation of A. potential energy and B. temperature in molecular dynamics trajectories. (JPG) [file pone.0062619.s001.jpg]
